# Study on the Properties of Alkali-Excited Concrete Modified by Nano-SiO_2_ Based on Response Surface Methodology

**DOI:** 10.3390/ma18102292

**Published:** 2025-05-15

**Authors:** Qiao Sun, Xin Wei, Renjie Cai, Dongwei Li

**Affiliations:** College of Civil Engineering and Architecture, Dalian University, Dalian 116622, China; sunqiao@dlu.edu.cn (Q.S.); weixin@s.dlu.edu.cn (X.W.); cairenjie19970708@163.com (R.C.)

**Keywords:** response surface method, microscopic characterization, nano-SiO_2_, alkali-activated concrete, mixture proportion optimization

## Abstract

To enhance the mechanical properties and low-carbon characteristics of industrial solid waste concrete, this paper proposes a synergistic modification strategy using nano-SiO_2_ and sodium silicate. The nano-SiO_2_ sol and sodium silicate activator were prepared using magnetic heating and stirring technology, and a quadratic regression model (R^2^ = 0.9575, *p* < 0.0001) for compressive strength with three factors and three levels was established using the response surface method (RSM-CCD). The modification mechanism was verified through optimization of the mix ratio using a desirability function, along with microscopic characterization via SEM and XRD. The results indicate the following: (1) the content of nano-SiO_2_ (2.4%) contributed the most to the compressive strength of the concrete, and its interaction with sodium silicate (2.1%) significantly promoted the formation of C-S-H gel; (2) the optimized fly ash substitution rate (21.7%) can achieve a 28-day compressive strength of 34.8 MPa, with the model prediction error controlled within 5%; (3) microscopic analysis showed that the synergistic effect of multiple components lowered the volume porosity of the cementitious phase, forming a densified network structure. The multi-factor synergistic optimization approach for nano-SiO_2_-modified alkali-activated concrete (NS-AAC) proposed in this study offers a reference for multi-objective mix design optimization of industrial waste-based concrete.

## 1. Introduction

With the acceleration of industrialization, the construction industry has become one of the primary contributors to resource consumption and greenhouse gas emissions. Statistics indicate that the global construction industry consumes approximately 4 billion tons of cement annually, with CO_2_ emissions from cement production accounting for over 8% of total global emissions [1]. To address environmental pollution and promote resource reutilization, fly ash, owing to its pozzolanic activity, is widely employed as a cement substitute. The synergistic use of nano-SiO_2_ and fly ash, combined with appropriate alkali activators, partially replaces cement in the production of nano-SiO_2_-modified alkali-activated concrete (NS-AAC). This approach not only effectively enhances the compressive strength of concrete but also significantly reduces the accumulation and landfilling of industrial waste, thereby conserving resources and protecting the environment [2]. However, fly ash concrete commonly exhibits defects such as rough surface texture, high water absorption, large porosity, and low early strength, which limit its engineering applications [3,4,5,6,7,8,9,10,11,12,13]. To expand the application scope of NS-AAC, comprehensive and systematic studies on its performance are necessary. Mix proportion design plays a decisive role in determining the workability, mechanical properties, and durability of concrete, serving as a critical prerequisite for performance optimization [14,15].

Currently, research on the mix proportion optimization of nano-SiO_2_-modified alkali-activated concrete (NS-AAC) remains limited. Most studies still refer to the “Code for Design of Ordinary Concrete Mix Proportions” [16] and employ orthogonal test methods to explore optimal mix ratios. However, orthogonal tests, when dealing with multi-factor mix proportions, often require a large number of experiments due to their inability to systematically consider interactions between variables, making it challenging to establish explicit functional relationships between factors and response values within a defined range [17,18]. In contrast, the response surface methodology (RSM), as a combination of mathematical and statistical techniques, can construct mathematical models between multiple factors and multiple response variables with fewer experimental runs, quantitatively analyze interactions among factors, and accurately predict optimal response conditions. RSM offers advantages such as high experimental efficiency, low cost, and high model fitting accuracy, making it particularly suitable for mix proportion optimization studies of multi-factor coupled systems like NS-AAC [19].

Previously, response surface methodology (RSM) was predominantly applied in fields such as biology, medicine, chemistry, agriculture, and food science. In recent years, it has gradually been adopted for optimizing mix proportions in cement mortar and concrete within the construction materials sector. Lu et al. [20] employed the Box–Behnken design (BBD) within RSM to establish a quadratic polynomial regression model, thereby determining the optimal mix proportions for ternary polymer mortar involving three factors and three responses. Wang et al. [21] developed a quadratic regression model using the BBD method within RSM to obtain the optimal mix proportions for high-strength coral aggregate concrete (CAC) involving three factors at three levels. Wang et al. [22] utilized the central composite design (CCD) within RSM to design experiments on basalt fiber foamed concrete and applied the desirability function for multi-objective optimization. Zhang et al. [23] employed the BBD approach within RSM to determine the optimal aggregate gradation and admixture dosage for recycled aggregate pervious concrete. These studies demonstrate the significant advantages of applying RSM for mix proportion optimization in construction materials; however, research utilizing RSM for nano-SiO_2_-modified alkali-activated concrete (NS-AAC) remains limited.

This study systematically investigates the mix proportion optimization of nano-SiO_2_-modified alkali-activated concrete (NS-AAC) using the response surface methodology with a central composite design (RSM-CCD). Initially, single-factor experiments were conducted to determine the optimal dosages of fly ash, nano-SiO_2_, and sodium silicate. Based on these results, factor levels for the response surface experiments were established. A quadratic regression model was then developed, with compressive strength as the response variable, to thoroughly analyze the effects of individual factors and their interactions on concrete performance. Subsequently, the optimal mix proportions for NS-AAC were determined using the numerical optimization function and desirability function in Design-Expert 13 software. Finally, the microstructure of the materials was characterized using scanning electron microscopy (SEM) and X-ray diffraction (XRD) to elucidate the modification mechanisms. This methodology not only accurately reveals the interaction patterns among factors but also provides robust support for the scientific mix design and multi-objective optimization of construction engineering materials.

## 2. Response Surface Method

**Figure 1:**
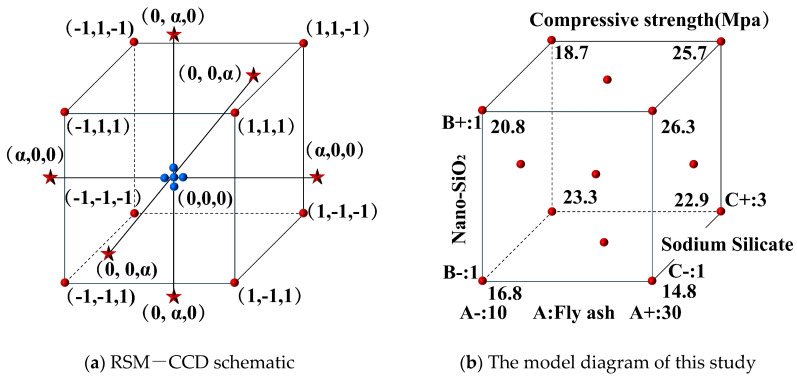
Three–dimensional schematic of the response surface model.

Response surface methodology (RSM) is a statistical technique employed to optimize processes and elucidate relationships among variables, aiming to identify optimal conditions or assess the influence of individual factors on response variables with a reduced number of experiments. Typical second-order response surface designs encompass Full Factorial Design (FFD), Box–Behnken design (BBD), and central composite design (CCD). Central composite design (CCD) offers a balanced approach regarding cost control, flexibility, accuracy, and applicability, rendering it particularly suitable for optimizing complex nonlinear models. Figure 1a illustrates the fundamental principles of RSM, wherein the incorporation of axial (star) and center points enhances the design’s flexibility, facilitating more accurate detection of quadratic effects and interactions, as depicted in Figure 1b. Taking into account the factor levels and the number of experimental runs in this study, a central composite design (CCD) within the RSM framework was employed to construct a three-factor, three-level regression model.

Response surface methodology (RSM) utilizes polynomial regression equations to model the relationships between factors and response variables. The standard procedure involves five key steps: collecting experimental data, constructing the response surface model, validating the model, optimizing the parameters, and verifying the optimal parameters. Equation (1) illustrates a polynomial response surface model comprising constant, linear, interaction, and quadratic terms.

(1) $$Y=\beta_{0}+\sum_{i=1}^{n}\left(\beta_{i}x_{i}\right)+\sum_{i=1}^{n}\left(\beta_{ii}x_{i}^{2}\right)+\sum_{1=i<j}^{n}\left(\beta_{ij}x_{i}x_{j}\right)+\varepsilon$$

where *Y* is the response variable, $\beta_{0}$ is the constant term, $x_{i}$ and $x_{j}$ are independent variables, $\beta_{i}$ is the coefficient of the linear term, $\beta_{ii}$ is the quadratic coefficient, $\beta_{ij}$ is the interaction term coefficient, ε is the random error, and n is the number of variables.

Analysis of variance (ANOVA) within the framework of response surface methodology (RSM) serves to evaluate the statistical significance of linear, interaction, and quadratic terms in predictive models. It also facilitates the assessment of regression coefficients and the overall model fit. The model’s reliability is quantified using the coefficient of determination R^2^ and the adjusted $R_{a}^{2}$, which accounts for the number of predictors relative to the sample size, as shown in Equations (2) and (3).

(2) $$R^{2}=1-\frac{S_{r}}{S_{m}+S_{r}}$$

(3) $$R_{a}^{2}=\frac{S_{r}/D_{r}}{\left(S_{m}+S_{r}\right)/\left(D_{m}+D_{r}\right)}$$

where *S_r_* represents the sum of squared residuals, *S_m_* denotes the regression sum of squares, *D_r_* refers to the degrees of freedom for the residuals, and *D_m_* corresponds to the degrees of freedom for the regression.

The Design-Expert 13 software incorporates a desirability function-based numerical optimization feature, enabling simultaneous determination of optimal variable levels and corresponding response values. The desirability function (*D*) ranges from 0 to 1 and quantifies the “desirability” of the response; the closer D is to 1, the more reliable the derived optimal conditions are. The individual desirability value (*Di*) and the expression of the desirability function are presented in Equations (4) and (5), respectively.

(4) $$D_{i}(y_{i})=\frac{y_{i}-y_{\min}}{y_{\max}-y_{\min}}$$

where y_min_ and y_max_ denote the minimum and maximum values of the response variable, respectively; y_i_ represents the current response value.

(5) $$D(y)=\prod_{i=1}^{k}D_{i}(y_{i})$$

where *k* represents the number of response variables; *D_i_*(*y_i_*) denotes the demand function for the individual response surface variable *y_i_*.

This study established the response surface regression model depicted in Equation (1) using 20 sets of compressive strength test data. Subsequently, the credibility of the regression model was evaluated through Equations (2) and (3). Finally, the optimal mix ratio for the experiment was selected by combining Equations (4) and (5).

## 3. Materials and Methods

### 3.1. Materials

The materials employed in this study adhere to the current Chinese national standards. The alkali activator utilized was instant powdered sodium silicate (commonly called “water glass”), exhibiting the same characteristics as its liquid counterpart and was procured from Zhengzhou Taichu Chemical Products Co., Ltd., Zhengzhou, China. Its physical properties are summarized in Table 1. Hydrophobic nano-SiO_2_ (NS ≥ 99.8%, 20 nm) was prepared by grafting dimethyldichlorosilane onto hydrophilic fumed silica (200 m^2^/g, specific surface area), supplied by Shandong Wanhua Tianhe New Materials Co., Ltd., Laiwu City, China. Its principal physical properties are listed in Table 2. First-class fly ash met the requirements of GB/T 1596-2005 (residue on a 5 μm sieve ≤ 18%) and was procured from Henan Hengyuan New Materials Co., Ltd., Henan, China. Its principal chemical composition appears in Table 3. P·O 32.5R cement (“Dalian Xinhu”) meets the standards of 3-day strength ≥ 15 MPa and a 28-day strength ≥ 32.5 MPa. Fine aggregate comprised dried, sieved natural river sand (0.15–4.75 mm), and coarse aggregate comprised sieved natural crushed gravel (5–20 mm), both conforming to standard grading specifications.

### 3.2. Sample Preparation Process

**Figure 2:**
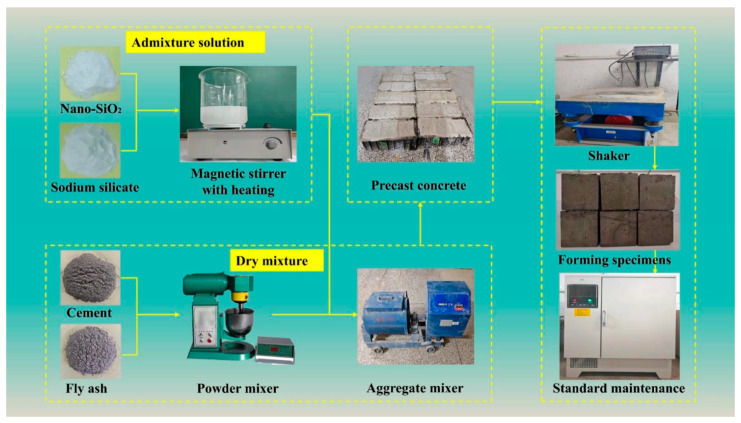
Flow chart of concrete specimen preparation.

The experimental study was conducted in accordance with the Standard for Test Method of Performance on Ordinary Fresh Concrete (GB/T 50080-2016) [24]. The concrete preparation process is illustrated in Figure 2. Initially, coarse and fine aggregates, fly ash, and cement were uniformly mixed and stirred for 30 s to form a dry mixture. Subsequently, sodium silicate and nano-SiO_2_ were separately mixed with water in proportion, and thoroughly stirred using a magnetic heating stirrer until completely dissolved, resulting in an alkali-activated solution and nano-SiO_2_ sol. Next, the prepared alkali-activated solution was added to the dry mixture, and stirring continued for an additional 30 s to establish an alkaline environment. Finally, the pre-prepared nano-SiO_2_ sol was incorporated, and stirring was maintained for 120 s to produce nano-SiO_2_-modified alkali-activated concrete. After mixing, the concrete was placed into cubic molds measuring 100 mm × 100 mm × 100 mm and vibrated on a vibration table for 150 s to achieve full compaction. Following vibration, the specimens were left to set for 24 h, demolded, and then cured for 28 days under conditions of 20 ± 2 °C and 95% relative humidity to obtain the newly prepared concrete specimens.

### 3.3. Experimental Method

#### 3.3.1. Compressive Strength Test Method

The compressive strength of prefabricated concrete cubes (100 mm × 100 mm × 100 mm) was tested using a YEW-33000 microcomputer-controlled pressure testing machine. The device is produced by Jinan Ruipu Electromechanical Technology Co., Ltd., Shandong, China. All specimens were tested after 28 days of standard curing at a constant loading rate of 2.4 kN/s. Each test group consisted of three parallel specimens, and the average compressive strength was used as the final result. The compressive strength was calculated using Equation (6).

(6) $$R_{c}=\frac{F_{C}}{A}$$

where *R_c_* is the compressive strength (MPa), *F_c_* is the maximum load at failure (N), and *A* is the loaded area (mm^2^).

#### 3.3.2. SEM and XRD Testing Methods

Thin slices (surface area ≤ 1 cm^2^) were extracted from concrete specimens after uniaxial compressive strength testing. First, the samples were immersed in absolute ethanol for 15 min to eliminate surface impurities. They were then dried in a 45 °C oven for 6 h. After natural drying, a 5 min gold sputter coating was applied to enhance conductivity for electron beam scanning. Finally, SEM imaging was performed using a ZEISS Gemini SEM500 (Carl Zeiss Microscopy Deutschland GmbH, Oberkochen, Germany) field-emission scanning electron microscope to observe the microstructure of the 28-day-cured modified and control concrete specimens.

Powder samples (≤1 g) were collected from concrete specimens after uniaxial compressive strength testing. The powders were homogenously dispersed and subjected to wide-angle X-ray diffraction (XRD) analysis. The scanning parameters were as follows: step size = 0.02°, dwell time = 0.24 s/step, and a 2θ range from 5° to 90°. All measurements were performed on 28-day-cured modified and control specimens to evaluate their phase compositions and crystalline structures.

## 4. Experimental Scheme

The experimental design was based on the “Code for Mix Proportion Design of Ordinary Concrete” [16]. In this study, 15 sets of single-factor experiments were conducted to determine the optimal dosages of sodium silicate, nano-SiO_2_, and fly ash. Specifically, the sodium silicate experiment comprised five groups with dosages of 1%, 2%, 3%, 4%, and 5% by mass of cement; nano-SiO_2_ was also tested in five groups with dosages of 1%, 2%, 3%, 4%, and 5%; and Class I fly ash was tested in five groups with cement replacement ratios of 10%, 15%, 20%, 25%, and 30%. The mix proportions of each component for the prefabricated concrete specimens are listed in Table 4, with a target concrete grade of C30. During specimen preparation, the amounts of water, fine aggregate, coarse aggregate, and superplasticizer were kept constant, while sodium silicate, nano-SiO_2_, and fly ash were replaced by percentages of cement mass. Additionally, cement, sodium silicate, nano-SiO_2_, and fly ash served as the binding materials, maintaining a water-to-binder ratio of 0.5 to ensure a consistent total binder content.

Based on the optimal dosage levels obtained from single-factor experiments, a three-factor, three-level response surface experiment was designed, selecting sodium silicate dosages of 1%, 2%, and 3%; nano-SiO_2_ dosages of 1%, 2%, and 3%; and fly ash replacement ratios of 10%, 20%, and 30%. The experimental data were statistically analyzed using the central composite design (CCD) function in Design-Expert 13 software. Table 5 presents the range of levels for each experimental factor, determined with reference to previous studies and the feasibility of mix proportions. In the experiments, fly ash, nano-SiO_2_, and sodium silicate are denoted as factors A, B, and C, respectively.

A total of 20 experimental runs were conducted in the response surface methodology, including 6 center point replicates, as detailed in Table 6. The compressive strength of the nano-SiO_2_-modified alkali-activated concrete specimens was tested after curing under standard conditions for 28 days.

Based on the optimal combination obtained from the response surface methodology, the compressive strength of concrete was optimized under these conditions using the numerical optimization function of the desirability function in Design-Expert 13 software. The goal was to maximize the compressive strength response (Y) of the concrete under these conditions. The optimization design objectives are presented in Table 7.

## 5. Experimental Results and Analysis

### 5.1. Analysis of Single-Factor Experimental Results

**Figure 3:**
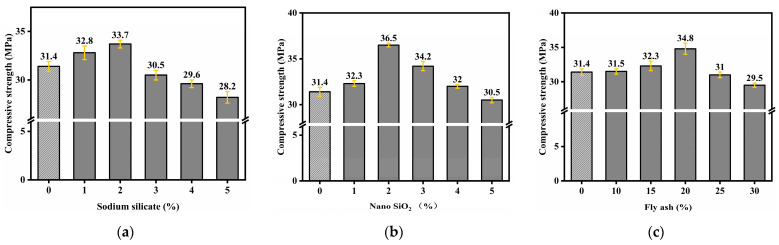
Compressive strength of alkali-excited nano-SiO_2_-modified fly ash concrete at different dosages: sodium silicate content (**a**), nano-SiO_2_ content (**b**), and fly ash content (**c**).

To investigate the effects of varying dosages of different admixtures on the compressive strength of concrete under standard curing conditions, Figure 3a illustrates that as the nano-SiO_2_ content increases, the compressive strength of the specimens initially increases and then decreases, remaining higher than that of the control group; the maximum compressive strength of 36.5 MPa is achieved at a 2% dosage. Figure 3b shows that as the dosage of the alkali activator increases from 1% to 5%, the compressive strength of the specimens initially increases and then decreases; a 2% dosage enhances the compressive strength, reaching a maximum of 33.7 MPa. Figure 3c indicates that replacing cement with fly ash within a certain range enhances the compressive strength of concrete; a 20% replacement rate yields the highest compressive strength of 34.8 MPa.

Nano-SiO_2_, due to its high specific surface area, can fill the pores in concrete and rapidly react with Ca(OH)_2_ to optimize the microstructure, thereby enhancing compressive strength. At low dosages, sodium silicate can activate reactive substances to form dense C-S-H gel; however, excessive amounts may trigger alkali-aggregate reactions, leading to increased porosity. Fly ash reacts with Ca(OH)_2_ through the pozzolanic effect to form cementitious products; when used in appropriate amounts, it serves both as a filler and strength enhancer, but excessive use can lead to insufficient hydration products and particle agglomeration, weakening the strength.

### 5.2. Optimization Design of Mix Ratio Based on Response Surface Methodology

The results of the response surface experiments are presented in Table 8. A response surface regression model was developed using 28-day compressive strength as the response variable, with fly ash (*A*), nano-SiO_2_ (*B*), and sodium silicate (*C*) as independent variables. The model, derived using Design-Expert 13.0 software, is expressed in Equation (7).

(7) $$Y=32.78+1.27A+1.72B+1.49C+1.85AB+0.4AC-2.15BC-5.76A^{2}-2.51B^{2}-3.36C^{2}$$

where Y represents the 28-day compressive strength (MPa); A denotes the dosage of fly ash; B denotes the dosage of nano-SiO_2_; and C denotes the dosage of sodium silicate.

An analysis of variance (ANOVA) was conducted on the response surface regression model, with the results presented in Table 9 and Table 10. The model (Y) exhibited a *p*-value less than 0.05, indicating high statistical significance and reliability. The individual factors A (fly ash replacement rate), B (nano-SiO_2_ dosage), and C (sodium silicate dosage), along with the interaction terms AB and BC, all had *p*-values less than 0.05, indicating significant effects on Y. Notably, interactions AB and BC had particularly pronounced impacts, whereas AC was not significant. Additionally, the quadratic terms A^2^, B^2^, and C^2^ were all significant. The difference between the adjusted R^2^ and predicted R^2^ was less than 0.2, the coefficient of variation (C.V.) was 6.25% (less than 10%), and the signal-to-noise ratio was 15.07 (greater than 4). The pure error was minimal, and the lack-of-fit test yielded a *p*-value of 0.4147 (>0.05), all indicating that the model has excellent fit and predictive capabilities, with stable and reliable experimental results. In summary, the model can accurately predict the effects of the selected factors on the compressive strength of nano-SiO_2_-modified alkali-activated concrete.

**Figure 4:**
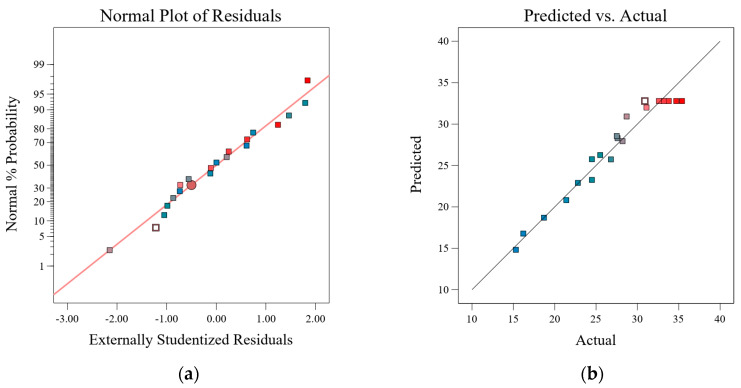
Diagram of the normal probability distribution of residuals (**a**); regression model actual vs. forecast plots (**b**).

Utilizing the ANOVA function in Design-Expert 13, a normal probability plot of residuals and a predicted versus actual values scatter plot were generated, as shown in Figure 4. In Figure 4a, the residual points closely align with the straight line, indicating that the model is feasible, exhibits linear correlation, and has a good fit. In Figure 4b, the predicted values closely match the actual values with minimal deviation, demonstrating that the regression model possesses high accuracy and adaptability.

**Figure 5:**
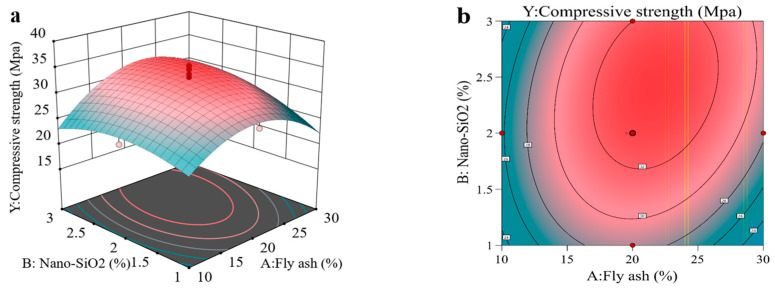

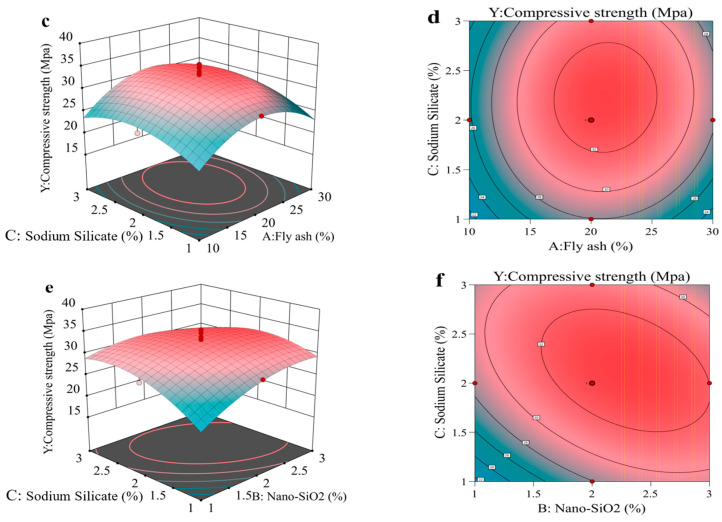
Compressive strength response surface plot with contour lines. (**a**,**b**) present the three-dimensional response surface and contour plots illustrating the interaction between factors A and B; (**c**,**d**) depict the response surface and contour plots for the interaction between factors B and C; (**e**,**f**) show the corresponding plots for the interaction between factors A and C.

The 28-day compressive strength response surface is shown in the figure, where three-dimensional response surface plots and corresponding contour plots illustrating the interaction effects of various factors were generated using Design-Expert 13 software. Figure 5a shows that the response surface of factors A and B exhibits significant curvature, indicating that the compressive strength Y of the concrete is sensitive to the interaction between A and B. When factor B remains constant, Y increases initially with A and then decreases; when factor A remains constant, Y shows a slight increase with B. The elliptical and dense contour lines in Figure 5b further confirm the significant interaction between factors A and B. Figure 5c indicates that in the interaction between factors A and C, the response of Y to A is greater than to C; the contour plot in Figure 5d shows the same trend. This is because the alkaline activator alone has limited activation of the cement hydration reaction, but when synergistically used with fly ash, it significantly enhances its pozzolanic reactivity, producing more hydration products. Therefore, under a constant dosage of the alkaline activator, the substitution rate of fly ash has a more significant effect on Y. Figure 5e shows that the surface curvature of the BC interaction is significant, with Y increasing as both B and C increase; the dense contour lines in Figure 5f also support this conclusion.

Based on the optimization design objectives and factor ranges for NS-AAC mix proportions, the optimal mix ratio and maximum compressive strength were obtained using Design-Expert 13 software. This corresponds to the optimal design results of the RSM-CCD method, as detailed in Table 11.

To further corroborate the accuracy of the RSM-CCD model, experiments were performed with the optimized NS-AAC mix proportions, resulting in an average 28-day compressive strength of 34.8MPa. Experimental results were compared against model predictions, and the absolute relative error (E) in compressive strength was computed according to Equation (8). As summarized in Table 12, all E values fell below 5%, satisfying the predictive-error threshold specified in GB/T 50081-2019 [25], which attests to the high predictive precision of the developed regression model. Therefore, employing the RSM-CCD regression model to optimize NS-AAC mix proportions not only enhances experimental efficiency but also facilitates the production of concrete exhibiting superior mechanical performance within a defined parameter range.

(8) $$E=\frac{\left|V_{T}{-V}_{P}\right|}{V_{T}}\times 100\%$$

where *E* is the absolute value of the relative error, *V_T_* is the test value of compressive strength, and *V_P_* is the predicted value of compressive strength.

### 5.3. Microscopic Characterization of SEM and XRD

#### 5.3.1. SEM Analysis

**Figure 6:**
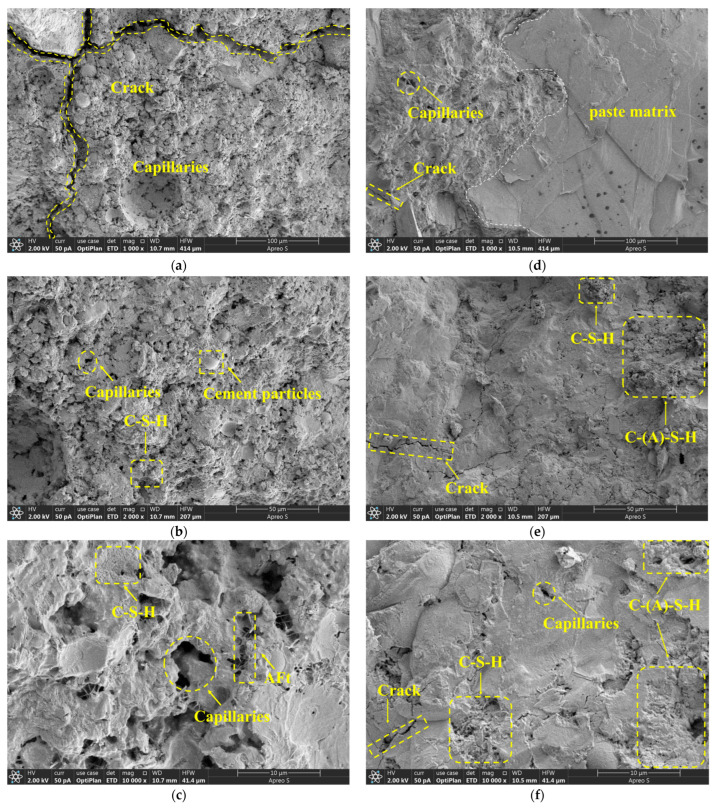
Concrete SEM comparison image: microstructural morphology of ordinary concrete (**a**–**c**); microstructural morphology of (**d**–**f**) modified concrete.

SEM images of the ordinary concrete (Figure 6a–c) reveal irregular macropores on the order of tens of micrometers under a 100 μm field of view, with clear detachment of sand particles from the cement paste at pore edges. Early-stage microcracks aggregate and extend in a network within the interfacial transition zone (ITZ). At 50 μm magnification, numerous submicron pores and partially unhydrated cement grains are observed. At the 10 μm scale, voids between needle- and plate-shaped hydration products act as stress concentrators for crack initiation. The overall high porosity and relatively weak aggregate–paste bonding suggest limited impermeability and crack resistance.

SEM images of the NS-AAC sample (Figure 6d–f) show a marked reduction in macropores under a 100 μm field of view, with most pore diameters below 5 μm, and aggregate–paste interfaces completely filled by dense gel phases. At 50 μm magnification, only minor cracks are observed, predominantly bridged by newly formed C–S–H/C–A–S–H gels exhibiting networked and dendritic morphologies that envelop and firmly bond coarse and fine aggregates. At the 10 μm scale, Ca(OH)_2_ plates are scarce, and nano-SiO_2_ particles are uniformly dispersed as nucleation sites, promoting the formation of short-fibrous or granular gels and dramatically reducing micropore sizes.

#### 5.3.2. XRD Analysis

**Figure 7:**
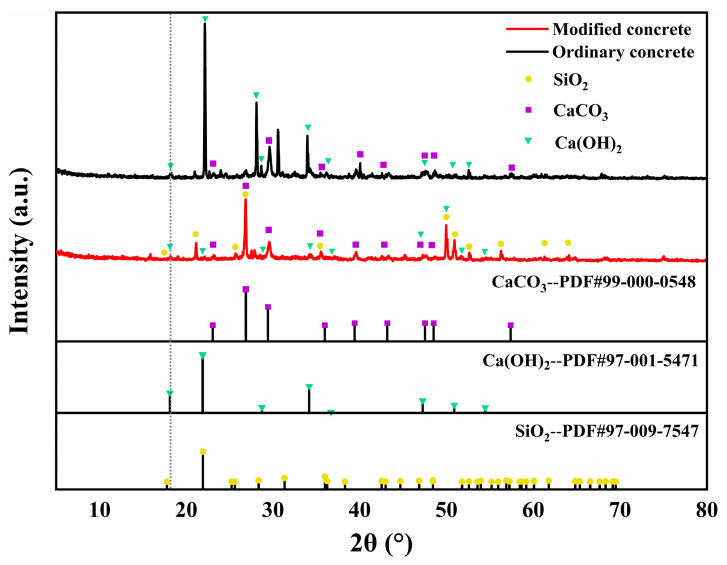
XRD spectra of ordinary concrete and modified concrete (NS-AAC).

Figure 7 presents the X-ray diffraction (XRD) patterns of ordinary concrete and NS-AAC after 28 days of curing. The ordinary concrete sample (black trace) exhibits pronounced diffraction peaks at 2θ ≈ 18.0°, 34.0°, and 47.1°, corresponding to the principal crystallographic planes of calcium hydroxide (Ca(OH)_2_). This observation indicates substantial formation of Ca(OH)_2_ crystals during cement hydration in the ordinary system. In contrast, the modified concrete (red trace) shows markedly attenuated peak intensities at these positions—most notably the primary peak at 2θ ≈ 18.0°—implying that the additive effectively engages in pozzolanic reactions with Ca(OH)_2_, thereby consuming free portlandite and promoting the formation of secondary C–S–H gel. Furthermore, a pronounced peak emerges at 2θ ≈ 26.6° in the modified sample, attributable to the quartz-type SiO_2_ phase, indicating the presence of reactive or inert silica components in the formulation. Simultaneously, enhanced peaks at 2θ ≈ 29.4°, 36.0°, and 39.4° correspond to CaCO_3_, likely arising from partial carbonation of portlandite or the introduction of additional carbonate phases.

In summary, the enhanced compressive strength of nano-SiO_2_-modified alkali-activated concrete can be attributed to the synergistic interplay between physical densification and chemical activation mechanisms. Firstly, the sodium silicate activator fosters primary hydration of the conventional cement matrix under highly alkaline conditions, thereby generating an increased amount of reactive Ca(OH)_2_. Subsequently, nano-SiO_2_ (NS) engages in pozzolanic reactions with free Ca(OH)_2_, both consuming deleterious portlandite and, via its nucleation effect, accelerating the formation of C–S–H/C–A–S–H gel phases. The ultrafine dimensions of NS further impart a pronounced filler effect, refining both macropores and microcracks, markedly reducing overall porosity and optimizing the gel network architecture. Moreover, the secondary activation of fly ash synergizes with NS to further promote the growth of amorphous gel, ultimately yielding a high-density, low-porosity microstructure that substantially enhances both mechanical performance and durability of the concrete.

## 6. Conclusions

This study systematically optimizes the mix design of nano-silica (SiO_2_)-modified alkali-activated concrete (NS-AAC) through response surface methodology based on a central composite design (RSM-CCD), in combination with advanced microstructural characterization techniques. The research elucidates the synergistic effects of multicomponent interactions on mechanical performance and microstructural evolution. The main findings are summarized as follows:

(1) The quadratic regression model developed for compressive strength (R^2^ = 0.9575, *p* < 0.0001) demonstrates high predictive reliability. Among all variables, nano-silica content at 2.4% exerts the most significant influence on strength (*p* = 0.0091), and its synergistic interaction with 2.1% sodium silicate (interaction term BC, *p* = 0.01) markedly enhances the formation of C-S-H gel.

(2) Multi-objective optimization identified the optimal mix as comprising 21.7% fly ash substitution, 2.4% nano-silica, and 2.1% sodium silicate. The 28-day compressive strength reached 34.8 MPa, with a prediction error of 4.5%, representing a 10.8% improvement over the control mix. Simultaneously, cement consumption was reduced by 21.7%, fulfilling the dual objectives of carbon reduction and mechanical enhancement.

(3) Microstructural analysis reveals that nano-silica reduces overall porosity through a dual mechanism involving physical pore filling (for pores < 5 μm) and chemical activation via Ca(OH)_2_ consumption, as confirmed by SEM observations. A denser C-S-H gel network is formed, corroborated by intensified quartz-phase SiO_2_ peaks in the XRD spectrum. Moreover, sodium silicate enhances the pozzolanic reactivity of fly ash, mitigates microcracking within the interfacial transition zone (ITZ), and suppresses the development of interfacial weakening phases.

## Figures and Tables

**Table 1:** Physical properties of instant powdered sodium silicate.

Index	Na_2_O Content	SiO_2_ Content	Modulus	Whiteness	Bulk Density
Detect	21.84%	60.71%	2.87	98.47%	0.56 g/cm^3^

**Table 2:** nano-SiO_2_ physical property parameters.

Index	SiO_2_	Specific Surface Area	Tap Density	PH of Suspension	Chloride	Al_2_O_3_	TiO_2_	Fe_2_O_3_
Detect	99.87%	125.00 m^2^/g	59.00 g/L	4.45	45.00%	12.00%	10.00%	7.00%

**Table 3:** Parameter table of physical properties of first-class fly ash.

Index	Al_2_O_3_	SiO_2_	SO_3_	CaO	Iron Content	Alkali Content	Water Content	Density	Bulk Density
Detect	36.80%	45.10%	1.20%	4.50%	0.85%	0.75%	0.40%	2.10 g/cm^3^	1.10 g/cm^3^

**Table 4:** The material mix ratio of each component of concrete (kg/m^3^).

Water	Cement	Fine Aggregates	Coarse Aggregates	Sodium Silicate	Nano-SiO_2_	Fly Ash	Water-Reducing Agent
210.0	420.0	680.0	1150.0	0	0	0	4.2
	369.6			4.2	4.2	42.0	
	340.2			8.4	8.4	63.0	
	310.8			12.6	12.6	84.0	
	281.4			16.8	16.8	105.0	
	252.0			21.0	21.0	126.0	

**Table 5:** Response surface test factors and levels.

Level	Factor		
	A (%)	B (%)	C (%)
−1	10.0	1.0	1.0
0	20.0	2.0	2.0
1	30.0	3.0	3.0

**Table 6:** NS-AAC mix proportion optimization experimental scheme (RSM-CCD).

Specimen Number	Variable Value			Coding Level		
	A (%)	B (%)	C (%)	A (%)	B (%)	C (%)
1	20	2	1	0	0	−1
2	20	1	2	0	−1	0
3	20	2	2	0	0	0
4	10	1	3	−1	−1	1
5	10	3	1	−1	1	−1
6	20	2	2	0	0	0
7	30	1	3	1	−1	1
8	20	2	2	0	0	0
9	20	2	2	0	0	0
10	30	3	3	1	1	1
11	30	2	2	1	0	0
12	20	2	3	0	0	1
13	20	2	2	0	0	0
14	30	3	1	1	1	−1
15	30	1	1	1	−1	−1
16	10	1	1	−1	−1	−1
17	10	2	2	−1	0	0
18	10	3	3	−1	1	1
19	20	2	2	0	0	0
20	20	3	2	0	1	0

**Table 7:** Mix optimization design objectives.

Parameters to Be Optimized	Range of Values		Target Value
	Min Value	Max Value	
A (%)	10.0	30.0	10.0–30.0
B (%)	1.0	3.0	1.0–3.0
C (%)	1.0	3.0	1.0–3.0
Y (MPa)	15.3	35.4	Maximum value

**Table 8:** Response surface test measurement and prediction.

Specimen Number	Factor			Compressive Strength (MPa)	
	A (%)	B (%)	C (%)	Measured Values	Predicted Value
1	20.0	2.0	1.0	28.2	27.9
2	20.0	1.0	2.0	27.5	28.1
3	20.0	2.0	2.0	33.2	32.8
4	10.0	1.0	3.0	24.5	23.3
5	10.0	3.0	1.0	21.4	20.8
6	20.0	2.0	2.0	35.4	32.8
7	30.0	1.0	3.0	22.8	22.9
8	20.0	2.0	2.0	30.9	32.8
9	20.0	2.0	2.0	32.6	32.8
10	30.0	3.0	3.0	26.8	25.7
11	30.0	2.0	2.0	27.6	28.3
12	20.0	2.0	3.0	28.7	30.9
13	20.0	2.0	2.0	33.8	32.8
14	30.0	3.0	1.0	25.5	26.3
15	30.0	1.0	1.0	15.3	14.8
16	10.0	1.0	1.0	16.2	16.8
17	10.0	2.0	2.0	24.5	25.8
18	10.0	3.0	3.0	18.7	18.7
19	20.0	2.0	2.0	34.7	32.8
20	20.0	3.0	2.0	31.1	32.0

**Table 9:** Analysis of variance of compressive strength regression model.

Data Sources	Sum of Squares	Degree of Freedom	Mean Square	F-Value	*p*-Value	Significant or Not
Y	640.63	9.00	71.78	25.05	<0.0001	Yes
A	16.13	1.00	16.13	5.68	0.04	Yes
B	29.58	1.00	29.58	10.41	0.01	Yes
C	22.20	1.00	22.20	7.81	0.02	Yes
AB	27.38	1.00	27.38	9.64	0.01	Yes
AC	1.28	1.00	1.28	0.45	0.52	No
BC	36.98	1.00	36.98	13.01	0.01	Yes
A^2^	91.92	1.00	91.92	32.10	0.0002	Yes
B^2^	17.31	1.00	17.31	6.09	0.03	Yes
C^2^	31.03	1.00	31.03	10.92	0.01	Yes
Residual	28.42	10.00	2.84	-	-	-
Lack-of-fit	15.64	5.00	3.13	1.22	0.42	No
Pure Error	12.77	5.00	2.55	-	-	-
Total Variation	669.04	19.00	-	-	-	-

**Table 10:** Regression model credibility test.

Model	Standard Deviation	Average	Correlation-R^2^	Adjustment-R_a_^2^	Prediction-R_p_^2^	Coefficient of Variation	Signal-to-Noise Ratio
Y	1.69	26.97	0.96	0.92	0.77	6.25%	15.07

**Table 11:** Design results of RSM-CCD mix optimization.

Mix Ratio Number	Fly Ash Content	Nano-SiO_2_ Content	Sodium Silicate Content	Compressive Strength	Desirability Function
Ⅰ	21.7%	2.4%	2.1%	33.3 MPa	0.9

**Table 12:** The optimal design results of the mix ratio were verified by the test.

*Y*		*E*
Predicted Value	Experimental Values	
33.3 MPa	34.8 MPa	4.5%

## Data Availability

The original contributions presented in this study are included in the article. Further inquiries can be directed to the corresponding author.
